# Effect of diet supplementation with chelated zinc, copper and manganese on hoof lesions of loose housed sows

**DOI:** 10.1186/s40813-016-0025-2

**Published:** 2016-02-10

**Authors:** Μarina Lisgara, Vassilis Skampardonis, Leonidas Leontides

**Affiliations:** Department of Epidemiology, Biostatistics and Economics of Animal Production, School of Veterinary Medicine, University of Thessaly, 224 Trikalon st., 43100 Karditsa, Greece

**Keywords:** Sows, Hoof lesions, Organic minerals

## Abstract

**Background:**

Hoof lesions are very common among sows and have been associated with lameness, early removal and compromised welfare and productivity. Although housing conditions and management can have an external effect on hoof health status, the role of trace mineral intake is vital in developing hoof structure and integrity. Therefore, the objective of this study was to investigate the effect of a diet supplemented with organic complexes of trace minerals (Zn, Cu, Mn), partly substituting their inorganic form, on hoof lesions of sows in three Greek swine herds.

**Results:**

A total of 518 sows were initially examined for hoof lesions and their respective severity was scored. For each hoof, the length of toes and dew claws were evaluated and five anatomical hoof sites, the heel, the sole, the white line, the wall and the coronary band, were examined for lesions. Subsequently, the same sows were re-scored after one or two gestations on diets supplemented with organic trace minerals, partly substituting their inorganic salt form (organic form of Zn 45 ppm, Cu 14 ppm and Mn 25 ppm of the total 125 ppm of Zn, 15 ppm of Cu and 40 ppm of Mn, respectively). The odds of the higher versus the lower lesion scores were significantly lower after than before the inclusion of the organic minerals in sows’ diet, for each of the considered foot sites with the exception of the coronary band, with a distinct effect according to foot location. Specifically, on rear feet the improvement of hoof lesions was either smaller (for heel, sole and wall) than on front feet or not significant (for white line, toe and dew claw length). Additionally, for each foot site and herd examined, after the inclusion of the organic minerals, there were more sows with either the same or lower lesion score, with the exception of the toe and the dew claw length in one of the herds.

**Conclusions:**

Within the specific conditions in the three studied herds, our findings highlight the role of chelated trace minerals in sows’ hoof health, suggesting an applicable and rewarding intervention to prevent hoof lesions.

## Background

The reported frequency of occurrence of hoof lesions of sows in modern swine herds is very high, usually exceeding 90 % of the studied sow populations [1–3]. Hoof lesions were associated with locomotor disorders [1, 3], early removal of sows [4, 5], decreased litter weight, decreased number of liveborn piglets and increased pre-weaning piglet mortality [6, 7]. Thus, hoof lesions hamper the achievement of sows’ optimum reproductive performance. Moreover, hoof lesions and the associated lameness could compromise sows’ welfare, due to the feeling of pain and subsequent locomotor impairment [8]. To promote sows’ welfare [9] the European Council (Directive 2001/88/EC) implemented the mandatory group housing of gestating sows in the European Union, since January 2013. However, group housing in sows was associated with increased leg and hoof disorders [1, 4, 6]. Despite the fact that housing conditions have an external effect on hoof health status [10], the role of trace mineral intake is vital for feet structure and integrity [11]. Keratinization of hoof epidermis is controlled and moderated by a variety of bioactive molecules and hormones. Minerals (Zn, Cu, Mn, Se, Ca) and vitamins (A, D, E and biotin) have a substantial contribution in production and preservation of healthy keratinized tissues [12, 13]. Particularly Zn, Cu and Mn were identified as key minerals in the process of keratinization [12, 14] since they have an instrumental role in the activation of enzymes with catalytic, structural and regulatory function [13, 15].

Increasing the bioavailability of trace minerals improves their utilization and thus may help improve the integrity of keratinized tissues in cattle [11, 16] and swine [17]. Many factors influence the bioavailability of trace minerals. The valence state of the mineral and its molecular form (inorganic or organic) in the diet are of major importance [18]. These specific properties of the mineral may be responsible for the complexes they form with other components in the gut, which may either obstruct or facilitate the mucosal absorption, transport and/or metabolism of the mineral in target tissues [18]. When conventional inorganic oxides and sulfates (e.g. ZnO, CuSO_4_) in feed break down in the stomach, the released ions are free to interact with ligands, which will either allow them to remain soluble in the intestine or bind them to insoluble chelates, like phytate, and form low solubility salts which are unabsorbable [19]. As a result, significant proportions of inorganic minerals are fecally excreted, stressing public concern about the environmental damage originated by intensive animal breeding operations [20]. To protect livestock, consumers and the environment the European Commission set maximum permitted levels for mineral concentrations in animal foodstuffs (Commission Regulation No 1881/2006) [21].

Current hyper-prolific sow genotypes have greater mineral demands than older genotypes, because they farrow and lactate larger litters [22, 23]. Thus, commercial sow diets are commonly formulated to exceed the NRC (1998) [24] mineral requirements [23]. Organic minerals, alternatively called chelated or proteinated minerals, are formed when the mineral is joined with an organic ligand, such as a protein or a specific amino acid [25]. Organic minerals are more soluble and thus can be absorbed by the intestinal lumen more easily compared to inorganic minerals [17], utilizing intestinal uptake processes of amino acids [26]. The biological advantage provided by organic compounds may be due to their unique coordination chemistry, which permits the formation of highly soluble, chemically stable products that resist digestion and interaction with antagonists in the gut [27, 28]. Therefore, using organic mineral sources instead of inorganic ones could help achieve a balance between animal trace mineral needs and their concentration in the diet, without affecting compliance with the limits of their inclusion rate in the feed [29].

Therefore, the purpose of this study was to investigate the effect of diet supplementation with chelated zinc (Zn), copper (Cu) and manganese (Mn) on hoof lesions of sows in three Greek swine herds.

## Results

A total of 518 sows, of which 124 were in herd A, 112 in herd B and 282 in herd C, were initially scored and subsequently re-scored after one (186/518, 35.91 %) or two (332/518, 64.09 %) gestations on diets supplemented with chelated trace minerals, as a partial substitution of their inorganic form. Upon initiation of the study, the median (range) parity of the studied sows was 1(1-8), 3(1-9) and 3 (1-6) for herd A, B and C, respectively. The proportion of sows with at least one lesion on any foot, at first scoring, was 120/124 (96.77 %), 110/112 (98.21 %) and 256/282 (90.78 %) in herds A, B and C, respectively. At second scoring 118/124 (95.16 %), 106/112 (94.64 %) and 250/282 (88.65 %) of sows in herds A, B and C, respectively, had at least one hoof lesion. By herd, in decreasing order, the four most frequently recorded lesions at first scoring were: In herd A, 112/124 (90.32 %) sows had at least one overgrown toe and an equal proportion of at least one overgrown dew claw [median of total lesion score 4 (range 0-8) and 3 (0-15), respectively], 56/124 (45.16 %) [median of total score 0 (range 0-8)] had at least one heel lesion and 50/124 (40.32 %) [median of total score 0 (range 0-7)] at least one wall lesion. In herd B, 100/112 (89.29 %) [median of total score 2 (range 0-10)] and 82/112 (73.21 %) [median of total score 1 (range 0-4)] sows had at least one heel or one wall lesion, respectively, while those with at least one overgrown toe or dew claw were, correspondingly, 80/112 (71.43 %) [median of total score 2 (range 0-7)] and 76/112 (67.86 %) [median of total score 2 (range 0-7)]. In herd C, sows with at least one heel lesion or one overgrown dew claw were 228/282 (80.85 %) [median of total score 4 (range 0-16)] and 198/282 (70.21 %) [median of total score 2 (range 0-16)], respectively, while those with at least one wall or one sole lesion were 120/282 (42.55 %) [median of total score 0 (range 0-8)] and 114/282 (40.43 %) [median of total score 0 (range 0-8)], respectively.

**Fig. 1:**
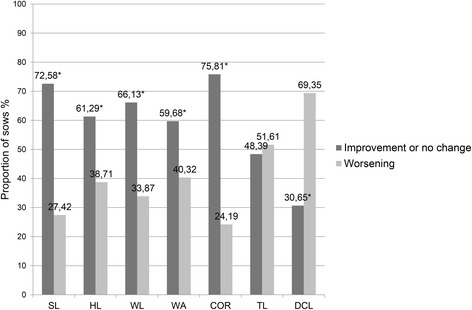
Frequency of improvement or no change and worsening in the severity of hoof lesions after diet supplementation with organic minerals, in herd A. The proportion of sows (n = 124) showing improvement or no change and worsening in the severity of lesions on SL (sole), HL (heel), WL (white line), WA (wall), CB (coronary band), TL (toe length) and DCL (dew claw length), after one or two gestations on a diet with organic trace minerals (Zn, Cu, Mn). Mc Nemar’s *χ* ^2^ test for symmetry was used to detect differences between the proportion of sows showing improvement or no change and the sows showing worsening in the severity of the hoof lesions, *P < 0.05

**Fig. 2:**
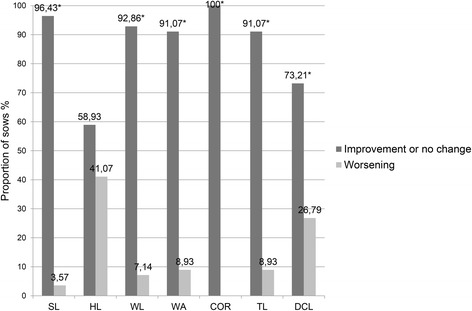
Frequency of improvement or no change and worsening in the severity of hoof lesions after diet supplementation with organic minerals, in herd B. The proportion of sows (n = 112) showing improvement or no change and worsening in the severity of lesions on SL (sole), HL (heel), WL (white line), WA (wall), CB (coronary band), TL (toe length) and DCL (dew claw length), after one or two gestations on a diet with organic trace minerals (Zn, Cu, Mn). Mc Nemar’s *χ* ^2^ test for symmetry was used to detect differences between the proportion of sows showing improvement or no change and the sows showing worsening in the severity of the hoof lesions, *P < 0.05

**Fig. 3:**
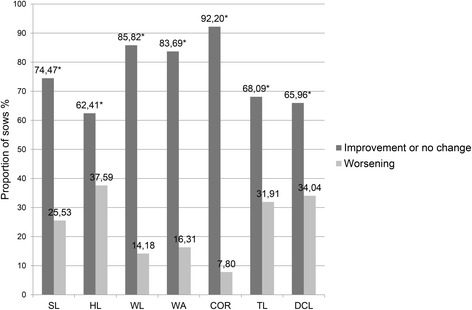
Frequency of improvement or no change and worsening in the severity of hoof lesions after diet supplementation with organic minerals, in herd C. The proportion of sows (n = 282) showing improvement or no change and worsening in the severity of lesions on SL (sole), HL (heel), WL (white line), WA (wall), CB (coronary band), TL (toe length) and DCL (dew claw length), after one or two gestations on a diet with organic trace minerals (Zn, Cu, Mn). Mc Nemar’s *χ* ^2^ test for symmetry was used to detect differences between the proportion of sows showing improvement or no change and the sows showing worsening in the severity of the hoof lesions, *P < 0.05

In Figs. 1, 2 and 3, the proportion of sows with improvement or no change in the severity (total score) of lesions on the different foot sites versus the proportion of those with lesion deterioration, after diet supplementation with organic trace minerals are presented by herd. In herd A, the proportion of sows showing improvement or no change in the severity of lesions on the sole, the heel, the white line, the wall and the coronary band, was higher (*P <* 0.05) than the one showing deterioration, whereas there was no difference (*P* = 0.72) for toe length. In contrast, for dew claw length, the proportion of sows with the same or lower score was lower (*P <* 0.001) than the one with higher score. In herds B and C, the frequency of improvement or no change in the total lesions score for all anatomical sites of the feet was higher (*P <* 0.001) compared to the frequency of deterioration; the only exception was the heel lesions of sows in herd B, where the before and after frequencies marginally did not differ (*P* = 0.06).

With the exception of the coronary band, final models for all other foot sites considered included an interaction between the dietary status and the foot. In addition, the final models for the severity of heel lesions and toe length included an interaction between the dietary status and the toe. In Table 1, the odds ratio for higher versus lower lesion severity score(s) (lesion score 2 vs score 1 or 0 and lesion score 2 or 1 vs score 0) for each foot site, are presented. Specifically, after the inclusion of the organic minerals in the diets, the odds of sole lesion score 2 versus 1 or 0 and score 2 or 1 versus 0 (higher versus lower score(s)), were 0.28 (95 % CI: 0.21, 0.38) and 0.48 (0.37, 0.61) times lower, for front and rear feet respectively, than before the inclusion of the organic minerals. Similarly, the odds of higher versus lower heel lesion score(s), after one or two gestations on diets with organic minerals, were 0.46- (95 % CI: 0.37, 0.57) and 0.69- (95 % CI: 0.55, 0.82) fold lower, for front and rear feet, respectively, and 0.34 (95 % CI: 0.27, 0.44) and 0.46 (95 % CI: 0.37, 0.57) times lower, for medial and lateral toes respectively, than before the inclusion of the organic minerals. The odds of higher versus lower white line lesion score(s) on sows’ front feet were 0.49 (95 % CI: 0.35, 0.69) times lower, after than before the inclusion of chelated minerals in the diets. However, there was no difference (*P* = 0.13) in the odds of higher versus lower score(s) of white line lesions on rear feet. For wall lesions, the odds of higher versus lower score(s) were 0.32 (95 % CI: 0.25, 0.42) and 0.50 (95 % CI: 0.40, 0.66) times lower on front and rear feet, respectively, after the inclusion of the organic minerals. For toe length, the odds of higher versus lower score(s) were 0.26-fold (95 % CI: 0.19, 0.35) lower for front feet and 0.36 (95 % CI: 0.28, 0.51) and 0.26 (95 % CI: 0.19, 0.35) times lower for medial and lateral toes respectively, after than before diet supplementation with organic mineral sources. We found no difference (*P* = 0.35) in the toe length score on rear feet before and after the inclusion of chelated minerals in sow diets. For the dew claw length the odds of higher versus lower score(s) after the inclusion of the organic minerals in the diets were 0.57 (95 % CI: 0.46, 0.70) times lower for front feet but there was no difference (*P* = 0.21) in the odds of dew claw length of rear feet. Lastly, there was no significant (*P* = 0.17) effect of the chelated mineral diets on the likelihood of lesions on the coronary band. For all hoof sites examined there was a significant (*P* < 0.05) association between the severity of hoof lesions and sows’ parity. Specifically, the odds of sole lesion score 2 versus 1 or 0 and score 2 or 1 versus 0 (higher versus lower score(s)), were 1.23 (95 % CI: 1.14, 1.33) times higher for one unit increase of sows’ parity. The odds of higher versus lower lesion score(s) were, 1.08 (95 % CI: 1.02, 1.15) and 1.12 (95 % CI: 1.02, 1.23) times higher for one unit increase of sows’ parity, for heel and white line lesions respectively. Similarly, for lesions on the wall and the coronary band the odds of higher versus lower lesion score(s) were 1.18 (95 % CI: 1.12, 1.27) and 1.16 (95 % CI: 1.03, 1.31) times higher, respectively, for one unit increase of sows’ parity. For toe and dew claw length, the odds of higher versus lower lesion score(s) were 1.15- (95 % CI: 1.06, 1.23) and 1.08- (95 % CI: 1.01, 1.16) fold higher, respectively, for one unit increase of sows’ parity.

**Table 1:** Associations between the severity of hoof lesions and the dietary status of the sows

Foot site	Foot location	Odds ratio^a^ (After/Before)	95 % CI	Toe location	Odds ratio^a^ (After/Before)	95 % CI
SL	Front	0.28*	0.21, 0.38	NA	-	-
	Rear	0.48*	0.37, 0.61		-	-
HL	Front	0.46*	0.37, 0.57	Medial	0.34*	0.27, 0.44
	Rear	0.69*	0.55, 0.82	Lateral	0.46*	0.37, 0.57
WL	Front	0.49*	0.35, 0.69	NA	-	-
	Rear	0.78	0.57, 1.07		-	-
WA	Front	0.32*	0.25, 0.42	NA	-	-
	Rear	0.50*	0.40, 0.66		-	-
TL	Front	0.26*	0.19, 0.35	Medial	0.36*	0.28, 0.51
	Rear	0.88	0.70, 1.13	Lateral	0.26*	0.19, 0.35
DCL	Front	0.57*	0.46, 0.70	NA	-	-
	Rear	0.88	0.74, 1.07		-	-

*SL* sole, *HL* heel, *WL* white line, *WA* wall, *TL* toe length, *DCL* dew claw length

Mixed-effect ordinal logistic regression models associating the severity of lesions (scaled from 0 to 2) on six foot sites of 518 sows, with a diet supplemented with organic trace minerals over a period of one or two gestations (After), following a conventional inorganic mineral source diet (Before), in terms of its interaction with the location of the foot (front or rear) or the location of the toe (lateral or medial)

^a^Odds ratios were adjusted for foot location (front or rear), toe location (lateral or medial), sows’ parity and farm

*NA* not applicable; the effect of the applied diet was not associated with toe location

*P < 0.05

## Discussion and conclusions

Previous studies have reported on the effect of flooring and management on the frequency and severity of hoof lesions of sows [30–32]. There are, however, sparse reports on the effect of nutrition on sow hoof lesions [17, 33, 34], with the exception of biotin supplementation [35, 36]. In studies conducted in dairy cows, replacing sulfate minerals with an organic source, decreased severity of white line lesions, sole ulcers and heel erosions [16, 37, 38]. Additionally, it was shown that the supply of a combination of complexed trace minerals is more beneficial to claw integrity than supplying a sole complexed trace mineral due to synergistic effects [39]. In this longitudinal study, we investigated the effect of gestation and lactation sow diets supplementation with organic complexes of trace minerals, over one or two gestations, on hoof lesions of sows, in three Greek swine herds with typical, at least to nationally applied standards, general management and housing. The average claw horn growth rate of sows was recently estimated at about 6.3 mm per month, whereas over the same period the overall mean wear rate was about 5.1 mm [40]. The average claw length of sows is about 5 cm [40]. Thus, a period of a reproductive cycle including one full-term gestation and four weeks lactation may be considered minimum for complete hoof horn regeneration.

Trace minerals have an important role in maintaining horn integrity [11, 13]. The use of chelated minerals could enhance their absorption and utilization in the body [19, 26], probably due to increased bioavailability [41, 42], and thus the integrity of keratinized tissues [16, 17]. More specifically, the benefit to hoof keratinization of adequate Zn, Cu and Mn supplementation is related with the reinforcement of the epidermal junctions with the corium, making them less susceptible to separation or breakdown, and ultimately the improved integrity of the epithelial tissue [39]. In general, our results suggest that chelated minerals have a beneficial effect on the health status of the majority of hoof anatomical sites and the severity of lesions. However, there were hoof sites on the rear feet which had no decrease in the risk of lesion severity.

The severity of hoof lesions is expected to increase by increasing sow parity [3]; this “parity-effect” was accounted for in our analysis. Therefore, interventions (managerial or nutritional) which result to a relatively stable or unaffected status of hoof lesions while sows carry out more gestations can be considered to have a positive effect [17]. We found that after inclusion of the chelated minerals, lesions on the heel, the sole and the hoof wall had decreased severity which was more pronounced on front than rear feet and on medial than lateral heel bulbs. It has been reported that lesions on these sites are more frequent and severe on rear than on front feet [2, 3, 43]. Moreover, the lateral heel bulb usually carries most of the sow’s weight and thus it is more prone to injuries compared to the medial heel bulb [1, 44, 45]. The white line is the junction of wall and sole horn. Lesions on this site, which were reported to be more frequent and severe on rear compared to front feet [43], corresponded to deep separation of the wall and sole junction. This separation may facilitate the invasion of bacteria into the corium, resulting in infection and inflammation. Therefore, since the reduction of the severity of sole and hoof wall lesions was slower on rear that front legs, our failure to demonstrate significant improvement on white line lesions may indicate that on rear feet, the diets supplemented with chelated trace minerals, may probably not avert, at least at the applied inclusion rate and/or time interval, the course or deterioration of such severe lesions. We have recently shown that sow laminitis may frequently occur [46]. We histologically examined claws of culled sows and recorded histological changes (e.g. hyperplasia, edema, hyperemia, hemorrhage, white blood cells), that had been previously described in cases of equine and bovine laminitis (i.e. inflammation of the lamellar corium) [47–50]. An inflammation in the corium may lead to interference in the supply of nutrients [51], since keratinocytes are dependent on receiving oxygen and nutrients from the fine microvasculature of the corium by diffusion across the basement membrane [12]. Moreover, we found a positive association between the existence of these pathological changes and higher total lesion score of the claw. These changes were more frequently observed on the rear feet and on the lateral toes where, we and others also found that hoof lesions are more frequent and severe [1–3], 43, 44]. Therefore, for hooves with more severe lesions (higher lesion score), which are more likely on rear feet and on lateral toes, there would be a greater interference in the supply of nutrients and, thus, their improvement may be a more demanding and/or time consuming process compared to the improvement of the respective lesion scores on front feet and on medial heel bulbs. Thus, for decrease in the risk of lesion severity on these sites, either the use of chelated minerals in the sow diets should be longer than the period investigated or these lesions on these sites are not reversible with the inclusion rate of chelated minerals investigated in this study. Lastly, the fact that the chelated minerals supplemented diets had no effect on the occurrence of lesions on the coronary band was most likely attributed to the very low frequency of lesions on this site (2.2 %, only 182 affected hooves in the totally 8288 examined, before and after the inclusion of chelated minerals in sows’ diet).

Overgrown toes and dew claws are two of the most frequently occurring hoof lesions [3]. When gilts are developing to sows, their legs tend to conform to the shape and size of toes, which is defined by the combined effect of genetic, managerial and nutritional factors. Thus, excessive toe length in sows may occur when gilts are not selected for proper feet development [52]. Because claw horn growth rate is greater than the respective wear rate, toe overgrowth could occur as a function of age, especially when sows are not provided with enough space for adequate exercise [40]. An older study suggested that routine hoof trimming may be a preventive measure against the development of hoof lesions [53]. More recent ones, did not recommend routine trimming for lesion reduction because they measured no clear effect on hoof lesion development and sow longevity; therefore the additional labor and costs associated with regular claw trimming were not justified [30, 31]. However, these studies did not combine nutritional interventions, such as inclusion of chelated Zn, Cu and Mn, with trimming. It may be that the use of organic minerals in sow diets prevents the development of hoof lesions on the heel, the sole and the hoof wall but offers less in the prevention of toe overgrowth, which may be affected by a variety of factors (i.e. genetic, housing, flooring, age) [54]. The length of dew claws, on the other hand, is also affected by genetic factors and age but not by housing and flooring because they do not normally touch the ground or bear any weight. In relevant reviews [11], [13] and published studies [17] no effect of trace minerals on dew claw overgrowth was suggested or found. Therefore, we also expected limited effect, if any, of the nutritional intervention on dew claws overgrowth. We have recently demonstrated that all hoof lesions, including overgrowths, act synergistically to impair the sow’s locomotor ability [3]. Therefore, in an attempt to collectively interpret the results of the present and previous studies we advocate routine trimming of overgrown toes [54] and dew claws, in herds where these lesions are frequent, in addition to proper gilt selection and use of organic minerals in sow diets for mitigation of the risk of sow feet problems.

The results of our study are limited to the extent that recording and scoring of lesions was conducted by farm personnel. Although there were training sessions for lesion characterization by the personnel, and the validity of a subsample of the recordings was verified by one of us (ML), there were differences among herds. These differences were due not only to the unavoidable imperfect validity and repeatability of personnel scorings, but also to the existing variations in management, productivity, and genetic lines of sows. Moreover, since data of our study originated from three herds, the generalization of our results to the national Greek sow herd is dangerous. However, the longitudinal nature of our study provided, as a within-subject analysis, an internal validity independent of random assignment and a substantial boost in statistical power [55]. Besides the high statistical efficiency, the employed intra-subject comparisons, with each sow acting as its own control, controlled for the influence of all possible confounders, genetic factors or traits that remained constant over time for each sow, automatically by design [56–58].

## Methods

### Study population

The study was carried out in three indoor, farrow-to-finish herds, with 330 (A), 160 (B) and 800 (C) sows, respectively, with Danbred (A, B) and Hermitage (C) genetics. For participation in the study the only criterion was the owners’ written consent. Neither the health status of the sows’ feet nor the frequency of locomotor disorders was considered for herd selection. During gestation sows were loose housed in static groups of eight to 12, with free-access to individual feed troughs in non-locking stalls, on combinations of concrete and slatted flooring. Pen design and flooring was in accordance to the requirements of the EU Directive for loose housing of gestating sows. All herds operated on weekly farrowing schedules.

### Study design

At the beginning of the study, the hooves of the sows were examined for lesions by three farm employees, one on each herd, upon their entry to the lactation facilities. Training of the employees to identify, characterize and evaluate the severity of hoof lesions was done by two of the authors (ML and LL). Training involved an initial session at the clinics of the School of Veterinary Medicine, University of Thessaly (Karditsa, Greece) where the different anatomical sites of the hoof were identified and representative hoof lesions collected in the slaughterhouse were characterized and scored. Afterwards, training on scoring hoof lesions was repeated on each farm and employees were provided with a collection of pictures and the video of the training material, for referencing. Each sow’s data were recorded on specially developed paper data-capture forms. The primary author visited all farms once a month, collected the completed data-capture forms, and cross-checked the data by re-examining a random sample of 20 % of the sows with the responsible farm employee.

Until first scoring, sows were raised and fed throughout their life with diets containing only inorganic sources of minerals. The diets were in meal form and contained inorganic sources of Zn, Cu and Mn (125 mg/kg feed Zn from ZnO, 15 mg/kg Cu from CuSO_4_ and 40 mg/kg Mn from MnO). Our nutritional intervention was the first occasion that the studied sows received a diet with an organic mineral source. Upon exit from the farrowing facilities the sows were offered gestation and later lactation diets in meal form supplemented with chelated trace minerals, from commercially available chelated mineral sources (Zinpro Performance Minerals®, Availa®Sow) as a partial substitution of their inorganic form (organic form of Zn 45 ppm, of Cu 14 ppm and of Mn 25 ppm of the total 125 ppm of Zn, 15 ppm of Cu and 40 ppm of Mn, respectively). From exit from the farrowing facilities until service, sows were offered a total of 4.0-4.5 kg daily of gestation feed. Thereafter, the daily amount of feed was 2.6-2.8 kg until 90 days and 3.2-3.5 kg from day 91 to 107 of gestation. The feed contained 12.6-12.8 MJ metabolizable energy (ME), 14.0-14.4 % crude protein, 6.2-6.4 g of digestible lysine, 15000 IU of vitamin A, 130 mg of vitamin E, 8.2-8.5 g of calcium (Ca), 5.2-5.5 g of phosphorus (P) and 0.45 mg of biotin per kg and was given either in one meal at 0700 h (Herd B and C) or split in half and offered in two meals at 0700 and 1600 h (Herd A). One week before the expected farrowing, sows were transferred to the lactation facilities where they were restricted fed until five days after farrowing and then were offered ad libitum typical lactation diets containing 13.5 MJ ME/kg dry matter and 16.5-17.0 % crude protein, 7.5-7.7 g of digestible lysine, 17000 IU of vitamin A, 180 mg of vitamin E, 8.9-9.2 g of Ca, 5.7-6.0 g of P and 0.45 mg of biotin. The re-evaluation of the sows’ hoof lesions was carried out after one or two gestations on diets supplemented with the organic trace minerals. For almost one third of the sows, re-scoring was performed at the first farrowing after the nutritional intervention, because they were not on farm for the second farrowing, whereas for the remaining sows re-scoring was conducted during the second farrowing after diet supplementation with chelated minerals.

The scoring system for hoof lesions applied in this study has been previously described in detail [3]. Briefly, for each hoof, five anatomical sites were examined: the heel (soft keratinized epidermis on the ventral surface of the hoof towards the caudal end), the sole (hard keratinized epidermis cranial to the heel on the ventral surface of the hoof, including the junction between heel and sole), the white line (junction between sole and wall), the wall (hard keratinized epidermis on the dorsal surface of the hoof) and the coronary band. All hooves were examined for the presence of cracks, erosions, ulcers, bruises, separation along the white line and hyper-keratinization and the respective anatomical sites were scored in a severity scale ranging from 0 to 2, where score 0 was given to hoof sites with no lesions or very small superficial ones. For the sole, score 1 was given to hooves with erosions and/or superficial heel-sole separation and 2 to hooves with ulcers and/or severe heel-sole separation. For the heel, score 1 was given to hooves with hyper-keratinization and erosions and 2 to hooves with hyper-keratinization and ulcers. For the white line score 1 was assigned to hooves with superficial separation and 2 to hooves with deep separation. For the wall, the score was 1 when cracks often accompanied by bruises were observed and 2 when deep cracks were noted. For toe and dew claw length, score 0 was assigned to toes and dew claws with normal length, score 1 to extended toes and dew claws touching the floor when the animal was standing and score 2 to overgrown and twisted or cracked toes and overgrown and twisted or crushed dew claws. For the coronary band the score ranged from 0 to 1 (0 = no lesions, 1 = lesions of any kind, edema, hemorrhage and/or necrosis).

### Statistical analysis

All statistical analyses were performed using Stata 13.1 (Stata Statistical Software. College Station, TX) and evaluated for significance at the 5 % level. The total score for the four feet for each anatomical site considered was obtained by adding the respective scores of each of the five hoof sites, toes and dew claws. Therefore, for all anatomical sites except the coronary band, the total score for the four feet could range from 0 to 16; for the coronary band, the total score varied between 0 and 8. Comparison of the proportion of sows with the same or lower total lesion score for each hoof site with the proportion of sows with higher score, before and after one or two gestations on diets supplemented with the organic trace minerals was performed by Mc Nemar’s *χ* ^2^ test for symmetry.

The possible effect of the dietary intervention with organic trace minerals on the severity of lesions on each hoof site considered, was estimated in seven mixed-effect, either ordinal (for all hoof sites except for the coronary band) or binary (for the coronary band), logistic regression models in GLAMM [59]. These models included the score of the anatomical site considered as the dependent variable and the dietary status (before or after the supplementation of the diet with organic trace minerals), the foot (front or rear), the toe (medial or lateral), the sow’s parity and the farm of sow’s origin as the independent variables. The latter two variables were forced in the models in order to control for their likely confounding effects. Furthermore, these models included a random-effect term for sow, a random-effect term for foot nested within sow and a random-effect term for toe nested within foot in order to account for the hierarchical structure of multiple measurements and repeated scoring on the same animal, foot and toe. The proportional odds assumption for the fitted ordinal regression models was verified graphically, by assessing the plots of the resulting empirical cumulative logit functions of each of the dependent variables considered. After initial fit of the models to the data all possible two-way interactions between the fixed effects for diet, foot and toe were created, were then offered one-by-one to the initial models and evaluated for significance at the 5 % level. The final models contained the fixed-effects for diet, foot and toe, the significant two-way interactions between the fixed effects and the random-effects.

### Ethics

This study was conducted in farms that complied with the current laws concerning the protection of animals kept for farming in the European Union [60]. Approval of the study protocol by an animal care committee was not required because taking part in the study was in no way painful or invasive for the animals.
